# Increased thiol levels in antimony-resistant *Leishmania infantum* isolated from treatment-refractory visceral leishmaniasis in Brazil

**DOI:** 10.1590/0074-02760170289

**Published:** 2018-02

**Authors:** Lucas S Magalhães, Lays GS Bomfim, Sthefanne G Mota, Geydson S Cruz, Cristiane B Corrêa, Diego M Tanajura, Michael W Lipscomb, Valéria M Borges, Amélia R de Jesus, Roque P de Almeida, Tatiana R de Moura

**Affiliations:** 1 Universidade Federal de Sergipe, Hospital Universitário, Laboratório de Biologia Molecular, Aracaju, SE, Brasil; 2 Howard University, Department of Biology, Washington DC, United States of America; 3 Fundação Oswaldo Cruz-Fiocruz, Instituto Gonçalo Moniz, Salvador, BA, Brasil

**Keywords:** Leishmania, visceral leishmaniasis, antimony, drug resistance

## Abstract

**BACKGROUND:**

Treatment-refractory visceral leishmaniasis (VL) has become an important problem in many countries.

**OBJECTIVES:**

We evaluated the antimony-resistance mechanisms of *Leishmania infantum* isolated from VL patients refractory or responsive to treatment with pentavalent antimony.

**METHODS:**

Strains isolated from antimony-refractory patients (*in vitro* antimony-resistant isolates) and antimony-responsive patients (*in vitro* antimony-sensitive isolates) were examined. Morphological changes were evaluated by transmission electron microscopy after trivalent antimony exposure. P-glycoprotein (P-gp) efflux pump activity was evaluated using the pump-specific inhibitor verapamil hydrochloride, and the role of thiol in trivalent antimony resistance was investigated using the enzymatic inhibitor L-buthionine sulfoximine.

**FINDINGS:**

Antimony treatment induced fewer alterations in the cellular structure of *L. infantum* resistant isolates than in that of sensitive isolates. P-gp efflux activity was not involved in antimony resistance in these isolates. Importantly, the resistant isolates contained higher levels of thiol compared to the sensitive isolates, and inhibition of thiol synthesis in the resistant isolates recovered their sensitivity to trivalent antimony treatment, and enhanced the production of reactive oxygen species in promastigotes exposed to the drug.

**MAIN CONCLUSIONS:**

Our results demonstrate that isolates from patients with antimony-refractory VL exhibited higher thiol levels than antimony-sensitive isolates. This indicates that redox metabolism plays an important role in the antimony-resistance of New World VL isolates.

Visceral leishmaniasis (VL) is a serious, oft-neglected tropical disease resulting from *Leishmania* parasite infection, which can be fatal if left untreated (WHO 2010). Specifically, New World VL is caused by *Leishmania infantum*. The disease is largely caused by parasitic adaptive mechanisms in the presence of immune insufficiency that fails to control infection. Chemotherapy is the main form of treatment. Since the 1940s, antimony compounds have been the principal therapy for leishmaniasis; however, since the 1980s, cases refractory to pentavalent antimony have continued to increase in incidence and prevalence (Mishra et al. 2007).

Therapeutic failure can be due to several factors, including drug-resistant parasites and/or subtle deficiencies in host immune mechanisms. Although several VL drug resistance mechanisms are well described, variations in the genetic and transcriptomic profiles of individuals and clinical isolates result in differences in susceptibility and drug resistance (Ponte-Sucre et al. 2013). Unfortunately, few studies have examined the drug sensitivity of clinical isolates from New World VL, focusing instead on laboratory mutants, the prototypical species *L. donovani*, and isolates from the Old World (Croft & Olliaro 2011). Understanding the resistance mechanisms and characteristics of New World clinical isolates will facilitate the development of more effective therapies against them.

One of the main known mechanisms of parasitic antimony-resistance involves the ABC transporter superfamily (Ouellette et al. 1998, Manzano et al. 2013). Most notably, the 170-kDa membrane-bound protein P-glycoprotein [(P-gp), also known as multidrug resistant protein 1] is responsible for the direct efflux of antimony compounds to the extracellular side of the membrane (Messaritakis et al. 2013). Thiol compounds are also involved in antimony resistance, by acting directly on toxic and free radical compounds in the intracellular environment, increasing parasite survival and proliferation (Singh et al. 2014). For instance, the increased expression of thiol pathway enzymes blocks antimony function by increasing thiol synthesis and subsequent drug conjugation, sequestration, and extrusion (Mukherjee et al. 2007).

Previous data from our group demonstrated that isolates from cases with VL relapse were resistant to antimonial compounds and nitric oxide. These isolates stimulated inflammatory cytokines and were resistant to macrophagekilling mechanisms, factors that may contribute to disease severity, but the mechanism underlying this observation was not clearly delineated (Santos et al. 2012, de Moura et al. 2015). Given the varied results of previous studies and the scarcity of publications on resistant parasites in the Americas, it is important to characterize the physiology of clinical isolates with intrinsic drug resistance. Thus, the aim of this study was to evaluate the mechanisms associated with antimony resistance of *L. infantum* isolates. This is the first study to evaluate clinical isolates obtained from antimony-refractory patients in the Americas.

## MATERIALS AND METHODS

*Parasites and culture conditions* - Clinical isolates from patients with VL were obtained by bone marrow puncture, before the start of the therapeutic regimen, and inoculated into Novy-MacNeal-Nicolle (NNN) and Schneider's Insect medium (Gibco, NY, USA) supplemented with 10% fetal bovine serum (Sigma-Aldrich Co., MO, USA) and 1% penicillin/streptomycin. The Ethics Committee of Brazil approved the project (CAAE-0151.0.107.000-07). Two isolates were obtained from antimony-refractory patients (*in vitro* antimony-resistant isolates, SbR), and two isolates were obtained from responsive patients (*in vitro* antimony-sensitive isolates, SbS). The clinical follow up of patients who were refractory to leishmanicidal drugs is described in table. Studied parasites were stored in frozen stocks after only one passage in culture, to avoid *in vitro* mutation. The patients were treated with meglumine, and those classified as partially improved received liposomal amphotericin B. *Leishmania* isolates were expanded in supplemented Schneider's medium at 24 ± 1°C. Promastigotes were examined daily using light microscopy to determine growth curves. The four isolates were tested to determine the half maximal inhibitory concentration (IC_50_; Table). Briefly, exponentially growing parasites (1 × 10^5^/mL) were treated with potassium antimonyl tartrate trihydrate (Sb^III^, 250-2000 μM; Sigma Chemical Co.) in 96-well plates for 48 h. Motile parasites were counted in a Neubauer chamber to determine the viable promastigotes at each concentration, as previously described (Santos et al. 2014).

**TABLE:** Clinical follow up with visceral leishmaniasis patients refractory and responsive to antimony treatment

Isolate	Year	Circumstances of sampling	Treatment	Clinical follow-up	Sb^III^ IC_50_ ± SD μM
SbS Isolate 1	2009	First episode	Meglumine	Improvement	253.3 ± 19.1
SbS Isolate 2	2009	First episode	Meglumine	Improvement	146.4 ± 24.9
SbR Isolate 1	2009	6th relapse	Meglumine	Partial improvement	804.2 ± 193,7*
SbR Isolate 2	2010	3th relapse	Meglumine	Partial improvement	752.3 ± 126.4*

Selected strains for studies: two isolates from antimony responsive patients (*in vitro* antimony sensitive isolates, SbS) and two from antimony refractory patients (*in vitro* antimony resistant isolates, SbR). IC_50_ previously determined after promastigote exposition to increasing concentrations of SbIII *in vitro*. Significant differences were determined using Student's *t*-test

* (p < 0.001).

*Transmission electron microscopy (TEM)* - Promastigotes in late exponential growth phase were washed with phosphate-buffered saline (PBS; Gibco, NY, USA) at 1620 × *g* for 10 min at 4°C, resuspended to 1 × 10^8^/mL in Schneider's medium (Sigma-Aldrich Co., MO, USA), and incubated for 48 h at 24°C in the presence or absence of 615 µM Sb^III^ (the average of the IC_50_ concentrations of all tested parasites). After exposure, the cells were washed and then fixed in a solution of 0.1 M sodium cacodylate buffer. The samples were subsequently post-fixed in 0.1 M cacodylate buffer with 1% osmium tetroxide and 0.8% potassium ferricyanide, dehydrated in an acetone gradient, and gradually embedded in Poly/Bed® (Polysciences Inc., PA, USA) prior to sectioning and staining with uranyl acetate and lead citrate. Morphological changes in the parasites and their organelles were observed and processed using a JEOL 1230 Transmission Electron Microscope (JEOL Ltd., Tokyo, Japan). The images were analysed in a qualitative and blinded fashion by two observers.

*P-glycoprotein-like transport pump activity analysis* - Rhodamine 123 (Thermo Fisher Scientific, MA, USA) is a fluorescent probe that can freely enter cells by passive diffusion, and its efflux is dependent on P-gp-type transport pumps (Forster et al. 2012). Verapamil hydrochloride (Sigma-Aldrich Co., MO, USA) is a classic inhibitor of P-gp-type pumps (Essodaïgui et al. 1999). Exponentially growing parasites (1 × 10^6^/mL) were washed with cold PBS, centrifuged at 1620 × *g* for 10 min at 4°C, and resuspended in 500 µL Schneider's medium containing 2.5 µM Rhodamine 123 with or without 100 µM verapamil hydrochloride, as previously described (Rai et al. 2013). The cells were incubated for 1 h in a darkroom, and then washed and resuspended in 500 mL PBS for analysis with a BD FACSCanto II flow cytometry system (Becton Dickson, NJ, USA).

*P-gp activity in Sb^III^-resistant isolates* - Efflux pump activity was examined as previously described, with some modifications (Neal et al. 1989, Valiathan et al. 2006). Briefly, promastigotes in exponential growth were washed, centrifuged at 1620 × *g* for 10 min at 4°C, then resuspended in Schneider's medium at 2 × 10^5^/well concentration. The isolates were then treated with Sb^III^ at their respective IC_50_ in the presence or absence of 8 µM verapamil hydrochloride for 48 h at 24°C. Motile parasites were counted in a Neubauer chamber to determine the concentration of viable promastigotes, as previously described (Santos et al. 2014).

*Determination of promastigote thiol levels* - Thiol levels in the promastigotes were determined according to a previously published protocol (Sarkar et al. 2009). Promastigotes in exponential growth were washed (1620 × *g*, 4°C, 10 min) and incubated with 1 µM CellTracker™ Violet BMQC fluorescent probe (Thermo Fisher Scientific, MA, USA), which binds to thiol components in the parasites with sufficient sensitivity to determine their concentration, for 20 min at 24°C. The probed cells were washed with PBS and then analysed by flow cytometry using a BD FACSCanto II.

*Viability analysis following treatment with Sb^III^ and a thiol synthesis inhibitor* - The ability of L-buthionine sulfoximine (BSO), an inhibitor of thiol synthesis, to reverse Sb^III^ resistance was determined *in vitro* using an adapted protocol (Kapoor et al. 2000). Promastigotes in the log phase of growth were washed (1620 × *g*, 4°C, 10 min), resuspended in Schneider's medium to 2 × 10^5^/well in 96-well plates, and then exposed to their respective Sb^III^ IC_50_ in the presence or absence of 5 mM BSO (Sigma-Aldrich Co., MO, USA) for 48 h at 24°C. Viability was determined by counting motile promastigotes in a Neubauer chamber.

*Reactive oxygen species (ROS) production after* in vitro *exposure to Sb* ^*III*^ *and BSO* - ROS were quantified after exposure to Sb^III^ as previously described by Fonseca-Silva et al. (2011). Promastigotes in the log phase of growth were washed (1000 × *g*, 4°C, 10 min), resuspended in Schneider's medium at 1 × 10^6^/mL in 96-well plates, and incubated with their respective Sb^III^ IC_50_ in the presence or absence of BSO for 48 h at 24°C. The cells were washed and then resuspended in PBS containing 25 µM 2′,7′-dichlorodihydrofluorescein diacetate (Sigma-Aldrich Co., MO, USA), a chemical component that is converted by oxidative reactions into a fluorescent component, and incubated for 30 min at 24°C. The fluorescent parasites were washed in PBS and analysed with a BD FACSCanto II flow cytometer.

*Statistical analysis* - Data represent the mean ± standard error of the mean (SEM). The normality of the data was analysed by Kolmogorov-Smirnov testing. Statistical analysis was performed using analysis of variance (ANOVA) with Tukey's post-test for parametric data, or the Kruskal-Wallis test with Dunn's post-test for nonparametric data. The analyses were conducted using GraphPad Prism 5.0 software (GraphPad Software Inc., CA, USA). Differences were considered statistically significant when p < 0.05.

## RESULTS

*Ultrastructural analysis of SbR and SbS L. infantum promastigotes* - SbR and SbS *L. infantum* isolates were treated with 615 µM Sb^III^ to evaluate ultrastructural changes by TEM. The treatment concentration was based on IC_50_ values determined by a dose-response curve (Table). In this curve, we tested a range of 250 to 2000 μM. At 500 μM, only 33% and 16% of the SbS isolates survived, while 70% and 67% of the SbR isolates survived. At 750 μM, only 30% and 13% of the SbS survived, while 57% and 42% of the SbR isolates survived. We used the same concentration of Sb^III^ for all parasite isolates to visualize the qualitative morphological modifications of susceptibility and resistance phenotypes after Sb^III^ exposure.

**Fig. 1:**
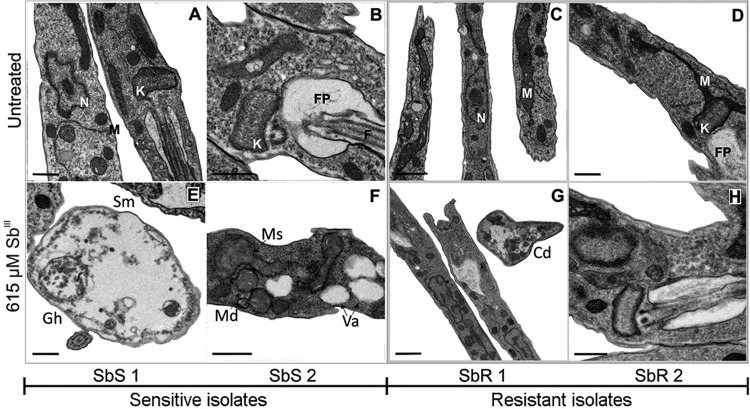
ultrastructural images obtained from transmission electron microscopy (TEM) in different conditions. Late log phase promastigotes were left untreated (A-D) or treated with 615 µM of Sb^III^ (E-H) for 48 h. Images are representative of the major alterations observed in each isolate and condition. SbR: antimony-resistant isolate; SbS: antimony-sensitive isolate. Ed: electrodensity; Gh: “Ghost” cells; Va: cytoplasmic vacuoles; Sm: absence of subpellicular microtubules; Ms: mitochondria swelling; Md: mitochondria disorganization. In the untreated parasites: N: nucleus; K: kinetoplast; M: mitochondria; FP: flagellar pocket; F: flagellum. Bars: A, B, D, E, F, H: 0.5 µM; C, G: 1 µM.

Untreated SbR and SbS isolates did not show any ultrastructural differences by TEM; however, 48 h exposure to Sb^III^ caused observable changes in all isolates, which were more evident in the SbS isolates. SbR isolates displayed changes in cell morphology, increased cytoplasmic electro density in the cell structure and cytoplasmic disorganization, and modifications associated with cellular adaptation to stress and sustained cellular viability. SbS isolates showed more intense cytoplasmic disorganization and vacuolization, an absence of subpellicular microtubules, mitochondrial cristae disorganization and swelling, and cells with a near absence of cytoplasmic content (“ghost cells”; Fig. 1A-H). These modifications are all indicative of decreased cell viability.

**Fig. 2:**
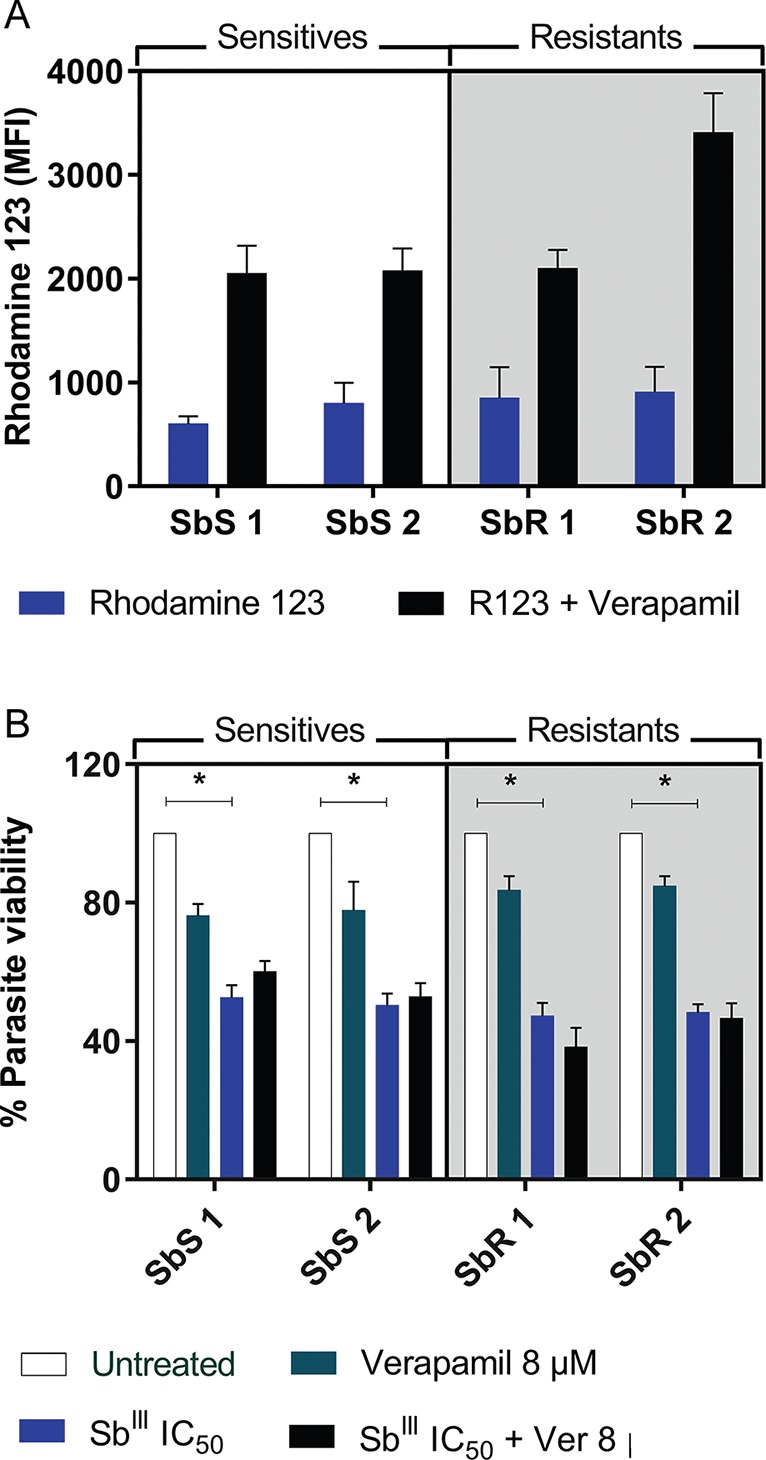
(A) rhodamine 123 uptake and accumulation mean fluorescence intensity (MFI) with or without verapamil blockade in *Leishmania infantum* promastigotes. Statistical analysis was performed by Kruskal-Wallis test. Results are the mean ± standard error of the mean (SEM) of three independent experiments. (B) Promastigote viability after exposure to Sb^III^ for 48 h with or without verapamil blockade. Each isolate was exposed to its own IC_50_. Statistical analysis was performed by ANOVA with Tukey's post-test. Statistical significance was defined in p < 0.05. Data shown are from two independent experiments performed in quintuplicate. SbS: antimony sensitive isolate; SbR: antimony resistant isolate.

*P-gp transport pumps do not directly influence antimony resistance* - ABC transporter activity has been reported to play a dominant role in drug efflux and sequestration in vesicles (Pérez-Victoria et al. 2001). To evaluate whether P-gp pump-dependent drug efflux was responsible for antimony resistance in these clinical isolates, cells were treated with the P-gp channel blocker verapamil hydrochloride. The blockade increased the concentration of intracellular Rhodamine 123, but this increase was not significant in any of the isolates. Additionally, no differences were observed between the resistant and sensitive isolates (p > 0.05; Fig. 2A). Moreover, when evaluating the efflux activity of P-gp channels in the antimony-resistant strains, no significant changes in promastigote viability were observed following *in vitro* exposure to Sb^III^ in the presence of verapamil (p > 0.05; Fig. 2B), suggesting that other resistance mechanisms are likely present.

**Fig. 3:**
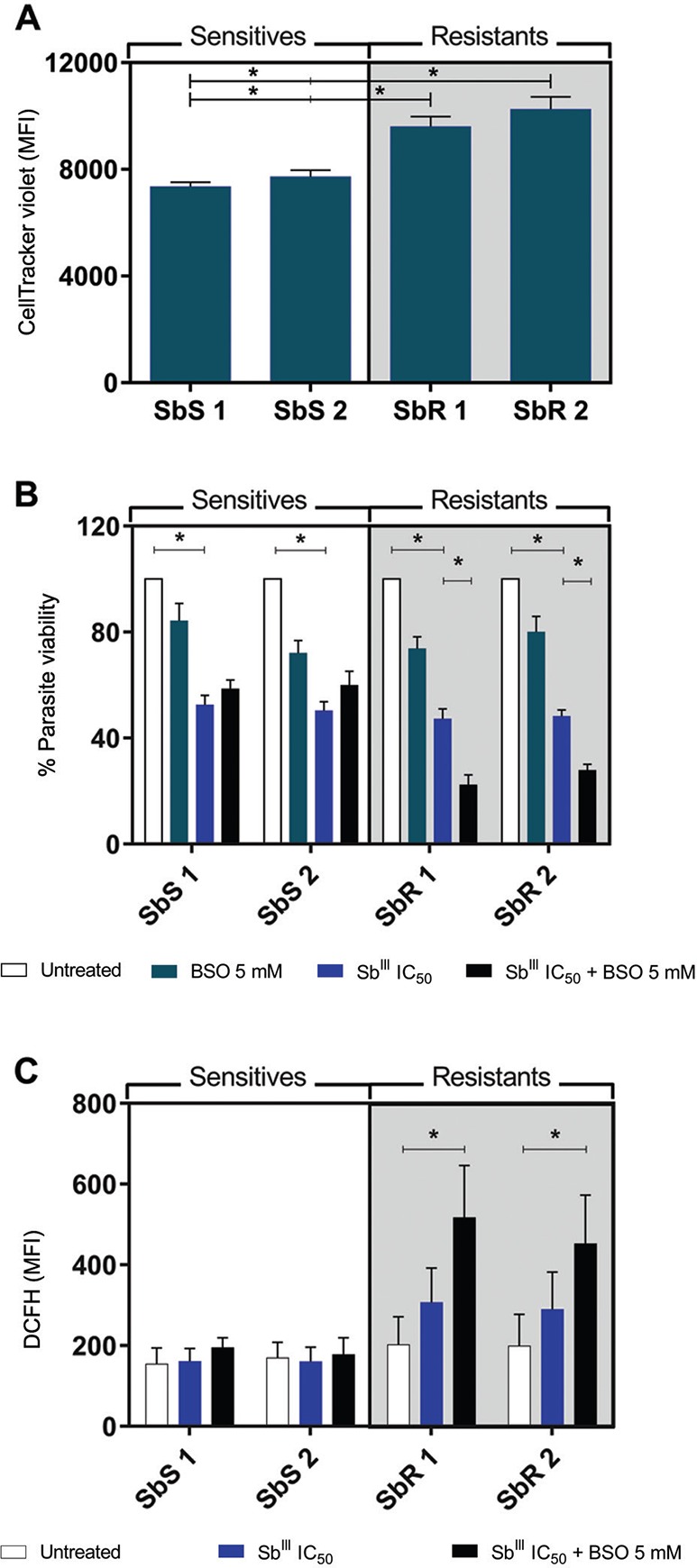
(A) thiol levels were measured (MFI) in promastigotes using CellTracker™ fluorescent probe (1 µM). Data are the mean ± standard error of the mean (SEM) of five independent experiments. (B) Parasite viability of promastigotes untreated and treated with Sb^III^ (at the IC_50_ of each isolate) in the presence or absence of L-buthionine sulfoximine (BSO). Data are the mean ± SEM of two independent experiments performed in quintuplicate. (C) ROS production after exposition to IC_50_ Sb^III^ with or without BSO measured by 2′,7′-dichlorofluorescein fluorescence (MFI). Statistical analysis was performed by ANOVA followed by Tukey's post-test. Statistical significance was defined as p < 0.05. Data represent the mean ± SEM of two independent experiments performed in quintuplicate. SbS: antimony sensitive isolate; SbR: antimony resistant isolate.

*Increased thiol levels contribute to antimony resistance* - Because increased thiol synthesis promotes antimony resistance, we quantified thiol levels in the resistant and sensitive isolates. Notably, compared to sensitive isolates, both resistant isolates exhibited approximately 1.24-fold higher thiol levels (p < 0.05; Fig. 3A). To assess whether this increase directly affected Sb^III^ resistance *in vitro*, promastigotes were exposed to the drug in the presence of the thiol synthesis inhibitor BSO. Exposure to their IC_50_ of Sb^III^ significantly reduced the viability of all isolates (p < 0.05; Fig. 3B); however, in the presence of BSO, resistant isolates were significantly less viable (p < 0.05), confirming that increased thiol levels are related to Sb^III^ resistance. Specifically, the parasite loads in resistant isolates 1 and 2 were 2.12 and 1.73-fold lower after exposure to Sb^III^ plus BSO compared to Sb^III^ alone. As the increased thiol metabolism in resistant isolates is known to potentiate the buffering capacity against ROS induced by antimony exposure (Mandal et al. 2007), we tested ROS production in these parasites after antimony treatment. We observed that resistant isolates produced similar amounts of ROS even when exposed to Sb^III^ concentrations 4-fold higher than those of sensitive isolates, further supporting enhanced thiol-mediated buffering capacity (Fig. 3C). Moreover, following a thiol synthesis blockade by BSO treatment, only SbR isolates exhibited a significant increase in ROS production (p < 0.05), confirming the role of increased thiol as a resistance mechanism to antimony and ROS-induced death.

**Fig. 4:**
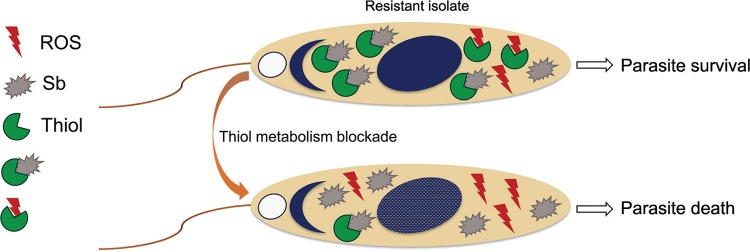
schematic model of the antimony-resistance mechanism in promastigotes of *Leishmania infantum* isolated from refractory patients from Brazil. In drug resistant isolates, higher thiol metabolism of thiol results in thiol-metal complex formation and drug inactivation. Additionally, the thiol component can buffer Sb^III^-induced ROS. Thereby, the parasites can survive at high concentrations of Sb^III^.

Taken together, these data suggest a model for the antimony resistance mechanism in promastigotes of *L. infantum* isolated from refractory patients from Brazil. In drug-resistant isolates, higher thiol metabolism results in the formation of thiol-metal complexes and drug inactivation. Additionally, the thiol component can buffer against Sb^III^-induced ROS, allowing parasitic survival at high concentrations of Sb^III^ (Fig. 4).

## DISCUSSION

Chemotherapy is the primary treatment regimen for leishmaniasis, because vaccines are not yet available. However, resistance and toxicity to treatment are major concerns. This study aimed to characterize antimony resistance mechanisms in isolates from patients with treatment-refractory VL in an endemic area of Brazil. Significantly, we found that high thiol metabolism is the mechanism explaining the antimony resistance of these *L. infantum* isolates.

We recently described antimony and nitric oxide cross-resistance in *L. infantum* isolates, which induces robust pro-inflammatory cytokine expression in macrophages (Santos et al. 2012, de Moura et al. 2015). However, the specific mechanisms underlying Sb^III^ resistance have not been clearly defined. The data in the present study strongly suggest that increased thiol levels can explain both resistance to antimony and nitric oxide in these parasites.

Resistance to antimonial compounds utilizes various metabolic pathways. These metabolic changes can lead to altered cellular morphology and structure (Borges et al. 2005). TEM analysis in the present study found that resistant promastigotes treated with Sb^III^ displayed modifications associated with cellular adaptations to stress, but maintained cellular viability, whereas sensitive isolates showed changes that clearly demonstrated the lethal effects of Sb^III^ on these isolates. Generalized changes were observed in the isolates, reinforcing the notion that antimony compounds act on metabolic pathways essential for parasite survival, as opposed to a single cellular structure or an organelle (Frézard et al. 2009). In addition, these findings reinforce the importance of cellular analysis at the ultrastructural level to fully characterize drug effects (Vannier-Santos & de Castro 2009).

Although the increased expression or activity of ABC superfamily transport pumps such as P-gp has been described as an antimony resistance mechanism, the present study showed no differences in transporter activity between resistant and sensitive isolates. Furthermore, no differences in parasite viability were observed with IC_50_ Sb^III^ treatment in the presence of the channel blocker verapamil hydrochloride, suggesting that another mechanism was likely responsible for antimony resistance. However, it is possible that a more sensitive method could detect effects of these pumps, albeit not as a major mechanism of resistance. More studies should be conducted to evaluate the relationship between efflux and antimony resistance. Parasites isolated from different geographic regions show diverse mechanisms of antimony resistance, and this heterogeneous pattern may be explained by the involvement of several different genes that have evolved to protect these parasites (Jeddi et al. 2014).

The present study demonstrates that the antimony resistance profile of the clinical isolates was primarily based on thiol availability. Notably, the resistant isolates displayed increased thiol expression compared to their sensitive counterparts. Furthermore, the inhibition of thiol synthesis by BSO treatment increased parasite sensitivity to Sb^III^. Previous studies have demonstrated the essential role of thiols in antimony inactivation (Rai et al. 2013). We observed that antimony-resistant promastigotes were also resistant to Sb^III^-induced ROS, which was enhanced by thiol synthesis inhibition, specifically in resistant isolates. Interestingly, our group previously demonstrated that *L. infantum* promastigotes and amastigotes show resistance to NO (Santos et al. 2012, de Moura et al. 2015). Additionally, previous studies indicate that trypanothione, a thiol molecule with antioxidant activity in *Leishmania* parasites, is able to sequester NO and maintain oxidative homeostasis (Bocedi et al. 2010). Moreover, antimony-resistant *L. donovani* parasites display more potent immune-modulating effects such as enhanced interleukin 10 expression in infected macrophages, which is associated with multidrug-resistant protein 1 overexpression and may contribute to disease severity (Mukherjee et al. 2013). Taken together, these data suggest that there is cross-resistance between Sb^III^ and ROS buffering capacity (Fig. 4). This reinforces previous evidence of an association between drug and microbicidal resistance mechanisms, and their association with increased parasite virulence is concerning because of the high prevalence of VL in Brazil (Martins-Melo et al. 2014), including the endemic area containing the patients in the present study, as our group recently showed (Campos et al. 2017). Moreover, previous studies indicate an increased likelihood of *in vitro* resistance in *L. infantum* clinical isolates from Brazilian HIV-positive patients (da Luz et al. 2011). In addition, continued ineffective treatment in humans and reservoirs may lead to the selection of resistant strains persistent in the environment (Seblova et al. 2014). These results demonstrate the need for a more complete characterization of these clinical isolates to further elucidate the mechanistic relationship between drug resistance and evasion from the microbicidal mechanisms of the immune system.

In conclusion, our results demonstrate that isolates from antimony-refractory patients with VL exhibited higher thiol levels than antimony-sensitive isolates. This is the first study with clinical isolates of *L. infantum* from patients refractory to treatment, and indicates that redox metabolism plays an important role in antimony resistance in New World VL isolates.
